# Retraction: MicroRNA‐612 inhibits cervical cancer progression by targeting NOB1

**DOI:** 10.1111/jcmm.18498

**Published:** 2024-06-19

Jin, Y., Zhou, X., Yao, X., Zhang, Z., Cui, M., Lin, Y. (2020), MicroRNA‐612 inhibits cervical cancer progression by targeting NOB1. Journal of Cellular and Molecular Medicine, 24: 3149–3156. https://doi.org/10.1111/jcmm.14985.

The above article, published online on 22 January 2020 in Wiley Online Library (wileyonlinelibrary.com), has been retracted by agreement between the journal Editor‐in‐Chief, Stefan N. Constantinescu, Foundation for Cellular and Molecular Medicine, and John Wiley and Sons Ltd. The retraction has been agreed following a report submitted by the authors. In their report, the authors state that, following further experimentation, they found that silencing MiR‐612 could not suppress tumorigenesis of cervical cancer in vivo. The authors have reported that although the results in vitro are similar to the previously published results, the differences between the results in vivo and in vitro fundamentally affect their confidence in the conclusions of this article. As such, all parties agree the article must be retracted.

